# The COVID-19 pandemic and nurses’ attitudes toward death*

**DOI:** 10.1590/1518.8345.4769.3448

**Published:** 2021-07-19

**Authors:** Maria Filomena Passos Teixeira Cardoso, Maria Manuela Ferreira Pereira da Silva Martins, Letícia de Lima Trindade, Olga Maria Pimenta Lopes Ribeiro, Esmeralda Faria Fonseca

**Affiliations:** 1 Universidade do Porto, Instituto de Ciências Biomédicas Abel Salazar, Porto, Portugal.; 2 Centro Hospitalar Universitário de São João, Direção de Enfermagem, Porto, Portugal.; 3 Universidade Fernando Pessoa, Departamento de Enfermagem, Porto, Portugal.; 4 Escola Superior de Enfermagem do Porto, Porto, Portugal.; 5 Centro de Investigação em Tecnologias e Serviços de Saúde, Grupo NursID, Porto, Portugal.; 6 Universidade do Estado de Santa Catarina, Departamento de Enfermagem, Santa Catarina, SC, Brazil.; 7 Universidade Comunitária da Região de Chapecó, Santa Catarina, SC, Brazil.; 8 Centro Hospitalar Universitário de São João, Serviço de Medicina, Porto, Portugal.

**Keywords:** Attitude to Death, Death, Nursing, Coronavirus Infections, Pandemics, Hospitals, Atitude Frente à Morte, Morte, Enfermagem, Infecções por Coronavírus, Pandemias, Hospitais, Actitud Hacia la Muerte, Muerte, Enfermería, Infecciones por Coronavirus, Pandemias, Hospitales

## Abstract

**Objective:**

to analyze nurses’ attitudes toward death in a hospital context after the critical period of the COVID-19 pandemic in Portugal.

**Method:**

this quantitative, descriptive, exploratory study was conducted in a university hospital and addressed 995 nurses. Revised Death Attitude Profile (DAP-R) was used to collect data, which were analyzed using analytical and inferential statistics.

**Results:**

the nurses most frequently agreed with the statements concerning the Neutral/Neutrality Acceptance and Fear. Age, marital status, profession, and unit of work influenced the nurses’ attitudes toward death. During the critical pandemic period, the nurses providing care to patients with COVID-19 presented the following means: Fear (28.89/±8.521) and Avoidance Acceptance (18.35/±7.116), which were higher than the mean obtained in the Escape Acceptance dimension, with significant differences (p=0.004).

**Conclusion:**

the nurses held Fear and Avoidance attitudes, revealing the need to qualify and support Nursing workers to cope with the death of those they provide care and manage pandemics and catastrophes.

## Abstract (pt)

**Objective:**

analisar as atitudes dos enfermeiros frente à morte no contexto hospitalar após o período crítico da pandemia por COVID-19 em Portugal.

**Método:**

estudo quantitativo, descritivo, exploratório, realizado em um hospital universitário, com a participação de 995 enfermeiros. Para a coleta de dados, utilizou-se a Escala de Avaliação do Perfil de Atitudes acerca da Morte, sendo os dados analisados por meio de estatísticas analítica e inferencial.

**Resultados:**

a concordância dos enfermeiros foi mais elevada nas afirmativas relativas às atitudes de Aceitação Neutral/Neutralidade e Medo. A idade, o estado civil, a categoria profissional e a área de trabalho foram variáveis que influenciaram as atitudes face à morte. Durante o período crítico da pandemia, os enfermeiros em atendimento à COVID-19 apresentaram as médias das atitudes Medo (28,89/±8,521) e Evitamento (18,35/±7,116) superiores em relação à atitude Aceitação como Escape, que apresentou diferenças significativas (p=0,004).

**Conclusão:**

os enfermeiros adotaram posturas de Medo e Evitamento, o que revela a necessidade de investir-se na qualificação e no apoio dos profissionais de Enfermagem, para o enfrentamento da morte daqueles que cuidam e o manejo das pandemias e catástrofes.

## Abstract (es)

**Objetivo:**

analizar las actitudes de los enfermeros frente a la muerte en el contexto hospitalario después del período crítico de la pandemia por COVID-19 en Portugal.

**Método:**

estudio cuantitativo, descriptivo y exploratorio, realizado en un hospital universitario, con la participación de 995 enfermeros. Para la recolección de datos se utilizó la Escala de Evaluación del Perfil de Actitudes acerca de la Muerte. Los datos fueron analizados por medio de la estadística analítica e inferencial.

**Resultados:**

la concordancia de los enfermeros fue más elevada en las afirmaciones relativas a las actitudes de Aceptación Neutral/Neutralidad y Miedo. La edad, el estado civil, la categoría profesional y el área de trabajo fueron variables que influenciaron las actitudes frente a la muerte. Durante el período crítico de la pandemia, los enfermeros que atendían el COVID-19 presentaron las medias de las actitudes: Miedo (28,89/ ± 8,521) y Evitación (18,35/ ± 7,116), superiores a las actitudes de Aceptación, como Escape, que presentó diferencias significativas (p=0,004).

**Conclusión:**

los enfermeros adoptaron posturas de Miedo y Evitación, lo que revela la necesidad de realizar inversiones en la calificación y en apoyo de los profesionales de Enfermería, a fin de hacer frente a la muerte de quienes cuidan y gestionan las pandemias y los desastres.

## Introduction

The COVID-19 pandemic changed our lives and brought about much uncertainty, changing the practice of workers, significantly altering the experiences and functioning of organizations^1^^-^ 2. In February 2020, recognizing that the pandemic situation in Europe and worldwide was challenging, Portugal started preparing and organizing the hospital addressed in this study to deal with the disease^3^ efficiently. Like other countries, decreasing the risk of infection and its spread were the primary objectives of this process^2^.

In the Portuguese context, the Directorate General of Health, Ministry of Health, and the government itself approved various guidelines, imposing the schools to interrupt its activities, prohibiting visitation and companions in hospitals and other public institutions, suspending the vacation of health workers, and providing orientations regarding the use of Personal Protective Equipment (PPE) appropriate to the various situations and services^4^.

Even though these guidelines enabled standardizing procedures in all Portuguese hospitals, successfully responding to the growing number of COVID-19 cases also depended on preparing competent and differentiated health care^3^. In this context, the hospital addressed in this study implemented adaptations to meet the community’s and workers’ needs, the epidemiological profile of cases, beds occupation and clinical guidelines as they emerged.

Considering the predictable increased need for highly complex care, the first measures implemented in many countries included providing medical inputs and equipment essential for health workers in the services involved and create, approve and disseminate standards and procedures^3^^-^ 5. Concomitantly, the needs of people in the different professions were verified, especially those of nurses, operational assistants and diagnostic and therapeutic technicians^3^.

After the first months in which the disease was disseminated, COVID-19 was characterized as a global emergency given its capacity to produce new cases and because it is a potentially fatal disease, considered the major pandemic of the last 100 years^6^.

As recently reported in the international literature, nurses were and are vital workers in the care process during the COVID-19 pandemic^1^^,^ 5 ^,^ 7, and their role has been emphasized in the surveillance, prevention, control of the virus spread, care provided to patients, research addressing the COVID-19, guidance provided to the community^8^ and in the reorganization of institutions.

The possibility of mobilizing nurses - many of whom were volunteers - from the services that decreased their activity to ensure care was provided to patients with COVID-19 was determinant to organize the hospital addressed in this study. The nurses’ ability to adapt to new challenges, comply with protective measures and respond to increasingly intensified emerging needs, was another critical factor of the strategy implemented^3^. The provision of nurses in the units assigned to patients with COVID-19 needed to be adjusted because safety demanded the teams to be reinforced. The fact is that dealing with complex care, assisting clinically unstable patients and frequently experiencing death situations culminate in workers becoming physically and mentally exhausted^8^^-^ 9.

The growing number of deaths caused by COVID-19 and its impact on health workers has been reported worldwide^8^^-^ 9. Even though death is an integral part of life^10^, this unknown virus and disease may elicit different responses from workers and it is essential to identify how health workers are coping with death. This is even more important among Nursing workers considering the long time these professionals spend with patients. Hence, the following question guided this study: what are the nurses’ attitudes toward death and associated factors during the COVID-19 pandemic, in a hospital context?

The death-dying process permeates Nursing practices; however, studies seldom address this topic, and discussions during nurses’ training are either restricted or fragmented^11^^-^ 13 while there is a lack of investment to better qualify these workers, especially for critical situations.

In this context, this study, which is part of a more extensive investigation addressing the topic since 2017, is intended to analyze nurses’ attitudes toward death in a hospital context after the critical period of the COVID-19 pandemic in Portugal.

## Method

This quantitative, descriptive, exploratory and cross-sectional study was conducted in a University Hospital Center (UHC) located in the north of Portugal.

This UHC was a referral hospital for COVID-19 patients. Its preparation included ensuring that PPE would be readily available, organizing the flow of patients with COVID-19 in hospitalization units and preparing units to receive patients who tested for the COVID-19 but were waiting for the results. The entire UHC was involved in providing care to these patients and those who sought the service due to other pathologies.

The medical unit (Medical Clinic in Brazil) allocated 129 beds in different services to admit patients with COVID-19. Another 30 beds were allocated but not used. The surgical unit, designated for urgent surgeries only, allocated 22 beds and the intensive care unit allocated 62 beds. Two other units, a medical and a surgical unit, each with 14 beds, were designated for patients who were waiting for the test results. Of the 900 beds available in the hospital, 241 (27%) were occupied in the critical period of the COVID-19 pandemic. All these wards were adapted for the treatment of patients with the pathology and with the support of the Infection Prevention and Control and Resistance to Antimicrobials Unit flows of movement of patients and “clean” and “contaminated materials” were created enabling the circulation of workers wearing PPE. The medical, surgical and intensive care units composed the study setting.

A sample of 995 participants was recruited from a universe of 1,345 nurses working in this hospital: 540 of whom provided care to patients with COVID-19 and the remaining worked in units providing care to patients who were not contaminated during admission or hospitalization. The inclusion criterion was providing care to adult inpatients in a medical, surgical and intensive care unit. Workers who were on any type of leave at the time of data collection (n=133) were excluded. Still, 217 nursing workers chose not to participate in the investigation.

During the critical pandemic period, from March 2nd to May 15th, 2020, the hospital totaled 6,758 urgent consultations, the cause of which was “SARS-CoV2 symptoms”, 5,576 of whom were discharged. Of the patients with COVID-19 seen in the emergency department, 1,800 were not hospitalized and monitored at home, while 398 patients were discharged after hospitalization. In the same period, 443 deaths occurred among patients without COVID-19 and 95 among inpatients with COVID-19. Of these, 69 died in the medical unit, 20 in the intensive care unit and six died in the surgical unit.

During data collection, the UHC had already returned to its regular activity and inpatients with the COVID-19 occupied only 33 beds (3.7%).

A self-report instrument addressing the nurses’ sociodemographic characteristics (sex, age, marital status, profession, field of specialization, unit of work, function in the unit providing care to patients with COVID-19, whether was absent from the hospital during the first critical period of the pandemic in Portugal) was used along with the Revised Death Attitude Profile (DAP-R), translated and adapted to the Portuguese population in 2010^14^. This instrument was composed of 32 close-ended questions rated on a seven-point Likert scale, ranging from 1 (strongly disagree) to 7 (strongly agree). The 32 items cover five dimensions: fear of death (7 items); death avoidance (5 items); neutral acceptance (5 items); approach acceptance (10 items) and escape acceptance (5 items). The total score ranges from 32 to 224.

The Fear dimension includes the items: 1 - Death is no doubt a grim experience; 2 - The prospects of my own death arouses anxiety in me; 7 - I am disturbed by the finality of death; 18 - I have an intense fear of death; 20 - The subject of life after death troubles me greatly; 21 - The fact that death will mean the end of everything as I know it frightens me; 32 - The uncertainty of not knowing what happens after death worries me.

The Avoidance dimension comprises the items: 3 - I avoid death at all costs; 10 - Whenever the thought of death enters my mind, I try to push it away; 12 - I always try not to think about death; 19 - I avoid thinking about death altogether; 26 - I try to have nothing to do with the subject of death;

The Approach Acceptance dimension includes items: 4 - I believe that I will be in heaven after I die; 8 - Death is an entrance to a place of ultimate satisfaction; 13 - I believe that heaven will be a much better place than this world; 15 - Death is a union with God and eternal bliss; 16 - Death brings a promise of a new and glorious life; 22 - I look forward to a reunion with my loved ones after I die; 25 - I see death as a passage to an eternal and blessed place; 27 - Death offers a wonderful release of the soul; 28 - One thing that gives me comfort in facing death is my belief in the afterlife; 31 - I look forward to life after death.

The Escape Acceptance dimension includes items: 5 - Death will bring an end to all my troubles; 9 - Death provides an escape from this terrible world; 11 - Death is deliverance from pain and suffering; 23 - I view death as a relief from earthly suffering; 29 - I see death as a relief from the burden of this life.

The Neutral/Neutrality Acceptance dimension includes items: 6 - Death should be viewed as a natural, undeniable, and unavoidable event; 14 - Death is a natural aspect of life; 17 - I would neither fear nor welcome it; 24 - Death is simply a part of the process of life; 30 - Death is neither good nor bad.

In this study, DAP-R presented good internal consistency with a Cronbach’s alpha equal to 0.869. It should be noted that, within the scope of the study, the instrument’s total score and its dimensions were considered the dependent variables, while the nurses’ social and occupational characteristics were the independent variables.

Data were collected after the critical period of the COVID-19 pandemic in the country, from May 15th to 31st, 2020. The indicator defining the infection transmissibility degree was stable in this period, with a mean smaller than one. That is, at a national level, each case infected originated less than a secondary case, on average. One of the authors collected the data. Questionnaires were delivered to all the nurses working in the units included in this study when they received clarification about the survey. The questionnaires were later collected on each unit at a date scheduled according to the workers’ availability. The questionnaires were composed of three parts: the first part presented and explained the study’s objectives, contained the Institutional Review Board’s authorization and provided information concerning the volunteering nature of participation, ensuring the application of ethical principles, among which, the participants’ anonymity; the second part addressed the participants’ sociodemographic information and the third part included the DAP-R.

Data analysis included descriptive and inferential statistics performed with Statistical Package for the Social Sciences (version 21.0). The quantitative variables were presented according to mean, median and standard deviation (±), with a 95% confidence interval. The distribution of data was verified using the Shapiro-Wilk test. To understand the distribution of variables and, considering their nature, the Kruskal-Wallis Test (Independent samples) and the Mann-Whitney U test were used with the level of significance adjusted by Bonferroni correction, equal to 5%/10 = 0.5%. The qualitative variables of interest were the nurses’ attitudes toward death, which were tested using Chi-square with the level of significance established at 5% (p<0.05) and expressed in absolute frequencies. The normality of data was tested using the Kolmogorov-Smirnov test before analyzing the influence of the sociodemographic variables and the attitudes of professionals working in services providing care to patients with COVID-19. As an integral part of a broader investigation, this study reflects only one component, carried out with statistical options different from the others.

The Institutional Review Board at the UHC approved this study (opinion report No. 102/2017), and the amendment proposal was approved in the review board’s meeting on May 29th, 2020. After receiving clarification regarding the study’s objectives, provided in the first part of the questionnaire, the nurses were free to either fill in the questionnaire or not and return it in a sealed envelop.

## Results

A total of 995 nurses participated in the study: 767 (77.1%) were women, aged 38.09 years old on average (± 8.965), minimum of 23 years old and maximum 65 years old. Regarding their marital status, 591 (59.4%) were married or in a stable union, 348 (35.0%) were single, 53 (5.3%) divorced and three (0.3%) were widowed.

Concerning their profession, 755 (75.9%) were general nurses, 219 (22.0%) had a specialization in different nursing fields, 21 (2.1%) were nurse managers. Of the specialist nurses, 102 (10.3%) had a specialization in Rehabilitation Nursing, 65 (6,5%) in Medical-Surgical Nursing, 26 (2.6%) in Community Nursing, 13 (1.3%) in Mental and Psychiatric Health, seven (0.7%) in Maternal and Obstetric Health and six (0.6%) were specialized in Child and Pediatric Health.

When asked about whether they worked in one of the units providing care to patients with COVID-19, 540 (54.3%) nurses answered affirmatively and 455 (45.7%) reported they did not work in these units. Of the 540 participants who worked in one of the units assisting COVID-19 patients, 217 (21.8%) were in the medical unit, 168 (16.9%) in the intensive care unit, 120 (12.1%) in the surgical unit, one (0.1%) in the emergency department and 29 (2.9%) worked in other areas such as screening and testing at home, controlling access by applying epidemiological survey and monitoring body temperature. Five (0.5%) of this group of participants did not report the unit where they worked, during the critical period of the COVID-19 pandemic.

Note that 91 (9.1%), out of the total participants, were on leave during March and April 2020:

As for the reasons for absence 30 (3.0%) were complying with the quarantine/prophylactic isolation, 19 (1.9%) were absent due to other diseases, 19 (1.9%) were providing family support, 17 (1.7%) had a COVID-19 diagnosis, four (0.4%) were on maternity leave while two (0.2%) participants were on vacation.

### Nurses’ attitudes toward death

Table 1 presents the results to support the analysis of nurses’ attitudes toward death in the context of the COVID-19 pandemic.

**Table 1:** Numerical and percentage distribution of the answers concerning nurses' attitudes toward death according to the items of the Revised Death Attitude Profile (n=995). Porto, Portugal, 2020

Items	Scores*						
	1	2	3	4	5	6	7
	n(%)						
Fear Attitude							
1	21(2.1)	101(10.2)	58(5.8)	173(17.4)	152(15.3)	297(29.8)	193(19.4)
2	39(3.9)	143(14.4)	67(6.7)	147(14.8)	201(20.2)	255(25.6)	143(14.4)
7	67(6.7)	191(19.2)	64(6.4)	317(31.9)	143(14.4)	165(16.6)	48(4.8)
18	90(9.0)	252(25.3)	116(11.7)	191(19.2)	177(17.8)	113(11.4)	56(5.6)
20	122(12.3)	316(31.8)	102(10.3)	318(32.0)	73(7.3)	41(4.1)	23(2.3)
21	55(5.5)	138(13.9)	59(5.9)	231(23.2)	221(22.2)	199(20.0)	92(9.2)
32	93(9.3)	196(19.7)	58(5.8)	307(30.9)	162(16.3)	134(13.5)	45(4.5)
Avoidance Attitude							
3	52(5.2)	221(22.2)	98(9.8)	220(22.1)	155(15.6)	170(17.1)	79(7.9)
10	88(8.8)	244(24.5)	125(12.6)	190(19.1)	145(14.6)	146(14.7)	57(5.7)
12	77(7.7)	246(24.7)	90(9.0)	218(21.9)	137(13.8)	165(16.6)	62(6.2)
19	76(7.6)	242(24.2)	104(10.5)	215(21.6)	152(15.3)	153(15.4)	53(5.3)
26	134(13.5)	366(36.8)	137(13.8)	239(24.0)	56(5.6)	51(5.1)	12(1.2)
Approach Acceptance Attitude							
4	135(13.6)	119(12.0)	23(2.3)	467(46.9)	79(7.9)	112(11.3)	60(6.0)
8	48(4.8)	167(16.8)	92(9.2)	559(56.2)	57(5.7)	57(5.7)	15(1.5)
13	146(14.7)	146(14.7)	41(4.1)	498(50.1)	53(5.3)	72(7.2)	39(3.9)
15	113(11.4)	91(9.1)	29(2.9)	554(55.7)	88(8.8)	86(8.6)	34(3.4)
16	161(16.2)	124(12.5)	27(2.7)	542(54.2)	51(5.1)	63(6.3)	27(2.7)
22	89(8.9)	109(11.0)	32(3.2)	495(49.7)	118(11.9)	117(11.8)	35(3.5)
25	117(11.8)	116(11.7)	34(3.4)	536(53.9)	64(6.4)	97(9.7)	31(3.1)
27	117(11.8)	132(13.3)	48(4.8)	562(56.5)	63(6.3)	50(5.0)	23(2.3)
28	67(6.7)	85(8.5)	27(2.7)	372(37.4)	171(17.2)	206(20.7)	67(6.7)
31	114(11.5)	221(22.2)	75(7.5)	431(43.3)	60(6.0)	66(6.6)	28(2.8)
Escape Acceptance Attitude							
5	325(32.7)	257(25.8)	45(4.5)	209(21.0)	39(3.9)	71(7.1)	49(4.9)
9	344(34.6)	318(32.0)	50(0.5)	221(22.2)	32(3.2)	21(2.1)	9(0.9)
11	89(8.9)	160(16.1)	87(8.7)	287(28.8)	191(19.2)	137(13.8)	44(4.4)
23	135(13.6)	214(21.5)	80(8.0)	302(30.4)	141(14.2)	91(9.1)	32(3.2)
29	218(21.9)	293(29.4)	81(8.1)	306(30.8)	51(5.1)	34(3.4)	12(1.2)
Neutral/Neutrality Acceptance Attitude							
6	4(0.4)	11(1.1)	8(0.8)	26(2.6)	118(11.9)	394(39.6)	434(43.6)
14	9(0.9)	5(0.5)	1(0.1)	32(3.2)	65(6.5)	436(43.8)	447(44.9)
17	34(3.4)	85(8.5)	62(6.2)	249(25.0)	140(14.1)	285(28.6)	140(14.1)
24	8(0.8)	11(1.1)	11(1.1)	65(6.5)	116(11.7)	479(48.1)	305(30.7)
30	28(2.8)	84(8.4)	51(5.1)	492(49.4)	104(10.5)	183(18.4)	53(5,3)

* Scores = 1 - Strongly disagree; 2 - Disagree; 3 - Moderately disagree; 4 - Neither disagree nor agree; 5 - moderately agree; 6 - Agree; 7 - Strongly agree

The analysis shows that most participants agreed with the items concerning Fear of death. Regarding the Avoidance dimension, in which individuals try not to think about death to decrease the stress such thoughts or feelings may elicit, most participants disagreed with the statements. Most participants neither agreed nor disagreed with the items concerning Approach Acceptance, which mainly focused on religious beliefs, that is, a belief that death meant being closer to God and having a happy life after death. Likewise, most participants neither agreed nor disagreed with the items concerning Escape Acceptance, in which death is seen as the end of pain and suffering. Finally, the items concerning Neutral/Neutrality Acceptance, in which death is seen as a natural part of life, compose the aspects in which the participants most frequently agreed or strongly agreed.

The scale’s total mean score was 126.97 (±21.928). Regarding attitudes toward death, the Approach Acceptance dimension obtained a mean equal to 37.16 (±11.675), followed by Fear, with a mean equal to 28.68 (±8,342), Neutral/Neutrality Acceptance with a mean equal to 27.33 (±3.825), Avoidance with a mean of 18.32 (±7.098) and finally, Escape Acceptance, with a mean equal to 15.42 (±6.010).

Then, attitudes about death and sociodemographic variables were analyzed, showing significant associations in Table 2.

**Table 2:** Analysis of significant of the components of attitudes towards death and the socio-occupational variables of the participants (n=995). Porto, Portugal, 2020

Significance*	DAP-R/Dimensions^†^					
	Total score	Fear	Avoidance	Approach Acceptance	Escape Acceptance	Neutral/Neutrality Acceptance
Age groups	0.004	0.511	0.910	0.002	0.000	0.670
Marital status	0.360	0.019	0.522	0.114	0.157	0.219
Profession	0.552	0.121	0.216	0.038	0.772	0.005

* Significance = Kruskal-Wallis test (independent samples)

† DAP-R = Revised Death Attitude Profile

Regarding age and its association with Approach Acceptance and Escape Acceptance, the participants aged between 36 and 45, between 46 and 55 and 56+ years old presented the highest medians highlighting the Acceptance as Escape Attitude with a higher median in the age group over 56 years.

Fear presented a different distribution according to marital status. Married participants presented a median higher than that obtained by divorced participants, who, in turn, presented a higher median than widowed participants.

Differences were found among the participants regarding the Approach Acceptance and Neutral/Neutrality Acceptance dimension according to their profession. Specialist nurses presented the highest median in the Approach Acceptance dimension, while nurse managers obtained the highest median in the Neutral/Neutrality Acceptance dimension.

Subsequently, following the analysis of the dimensions that integrate the different attitudes about death and work areas (Table 3), slight differences in the means were identified, which were higher in the Fear and Avoidance attitudes, in the group of participants who worked in service areas of COVID-19.

**Table 3:** Mean scores of attitudes toward death between two groups of participants (n=995). Porto, Portugal, 2020

DAP-R* Dimensions	Unit of work	Mean	Standard Deviation
Fear	COVID-19 unit	28.89	8.521
	Other unit	28.44	8.127
Death Avoidance	COVID-19 unit	18.35	7.116
	Other unit	18.29	7.084
Approach Acceptance	COVID-19 unit	36.73	11.781
	Other unit	37.66	11.540
Escape Acceptance	COVID-19 unit	14.94	6.033
	Other unit	15.98	5.940
Neutral/Neutrality Acceptance	COVID-19 unit	27.28	3.845
	Other unit	27.39	3.805

* DAP-R = Revised Death Attitude Profile

Even though differences were found in the means obtained by the participants working in the units providing care to COVID-19 patients, this group obtained the highest means in the Fear and Death Avoidance dimensions, while differences were significant in the Escape Acceptance dimension (Table 4). In this sense, the nurses working in these contexts obtained the lowest mean in the Escape Acceptance.

**Table 4:** Analysis of significance between the dimensions concerning attitudes toward death (DAP-R) and working in an unit providing care to COVID-19 patients (n=540). Porto, Portugal, 2020

DAP-R* Dimensions	Significance^†^
Scale's total score	0.214
Fear	0.454
Death Avoidance	0.870
Approach Acceptance	0.259
Escape Acceptance	0.004
Neutral/Neutrality Acceptance	0.919

* DAP-R =Revised Death Attitude Profile;

† Significance - Mann-Whitney U test (independent samples)

Afterward, we analyzed whether the sociodemographic and occupational variables of the nurses working in the units providing care to COVID-19 patients influenced their attitudes toward death. First, we verified the normality of data using the Kolmogorov-Smirnov test. The hypothesis of normality would be rejected for all the variables with a level of significance of 5%, which was confirmed for all the variables.

Consequently, as ANOVA could not be used to compare the means, the non-parametric test Kruskal-Wallis was used instead. All, but one of the results, were not significant; therefore, we assume equality of all means. For instance, we assume that the means concerning Fear are equal for all age groups, marital statuses and professions. Likewise, we assume it for all the other variables, except for the Escape Acceptance dimension regarding the age groups.

Thus, we conclude that there are differences between the means according to the age groups concerning the Escape dimension. The Mann-Whitney test was used to compare the means with the level of significance adjusted by Bonferroni correction, 5% (10 comparisons). The Escape Acceptance means obtained by the individuals aged between 46 and 55 were higher than the means obtained by those younger than 25 years of age, while the Escape average obtained by participants 56 years old or older was also higher than that obtained by those younger than 25 years old.

Analysis of the nurses’ unit of work (medical, surgical, or intensive care unit) using the Kruskal-Wallis test for independent samples revealed significant differences between the scale’s dimensions and the participants’ units of work (Table 5).

**Table 5:** Analysis of significance of association between the dimensions of attitudes toward death (DAP-R) and unit of work (n=995). Porto, Portugal, 2020

DAP-R* Dimensions	Significance^†^
Scale's total score	0.000
Fear	0.000
Avoidance	0.000
Approach Acceptance	0.274
Escape Acceptance	0.047
Neutral/Neutrality Acceptance	0.129

* DAP-R = Revised Death Attitude Profile;

† Significance = Kruskal-Wallis test for independent samples

The surgical unit obtained the highest medians in the Fear Avoidance, and Escape Acceptance dimensions, while the medical unit obtained the lowest means in the Fear and Avoidance dimensions.

## Discussion

When compared to other prevalent pathologies and clinical conditions, the COVID-19 pandemic resulted in considerably high mortality rates worldwide in a very short time. It is essential to identify attitudes toward death of the primary professionals working in the front line fighting the pandemic in this context. One study^15^ warned about the importance of understanding how working tasks and conditions contribute to disseminating the pathology and monitoring the strategies established. In addition to these aspects, these findings also show the importance of understanding how workers cope with the disease’s repercussions and what are the resources available.

Death was and still is one outcome of this disease with which health workers providing care have to deal^16^^-^ 17. This aspect enables understanding the results obtained by the DAP-R concerning the Neutral/Neutrality Acceptance dimension, in which most workers agreed with the statements concerning death being a natural part of life. Simultaneously, the remaining attitudes depending on external influences, such as personal beliefs and convictions, obtained more undefined responses; that is, the participants neither agreed nor disagreed with the statements.

Even though many of the answers remained undefined, that is, the participants neither agreed nor disagreed, the Approach Acceptance dimension obtained a high mean. The belief that death means being closer to a superior being is not always clear. Nonetheless, the participants aged between 36 and 45 and between 46 and 55 were more emphatic on their beliefs and convictions, obtaining the highest means in the Approach Acceptance dimension. Given their life philosophies, younger workers may be more resilient with the challenges imposed^1^^,^ 5. Participants 56+ years old, in turn, obtained the highest median in the Escape Acceptance dimension, that is, they consider death an end to suffering, considering that older patients are more likely to die due to the novel coronavirus. Additionally, attitudes may be associated with experiences and personal and professional knowledge when facing rapid and severe changes that may affect the general condition of individuals with the disease^5^.

Working with the care provided to patients with COVID-19 and witnessing suffering among those with the disease led nurses to experience tension and anguish^1^. The Escape Acceptance dimension was less evident among those who worked between March and April in the units providing care to patients with COVID-19 than among those who did not work in these units. Perhaps, because the disease progresses rapidly and the media regularly and widely disseminated news indicating the growing number of contaminated and dead individuals, the participants not always saw death as an end to suffering, which was often of short duration.

Studies^18^^-^ 20 sought to understand the multiple impacts of the pandemic on people and health workers’ lives. Among the aspects observed, some studies have debated the mental health of these workers, which indicates the difficulty of preparing to face the problem.

Still, regarding attitudes toward death, Fear of death was significant among married participants or those living in a stable union. It is believed that the risk of being a carrier of the disease with the potential to transmit it to family members was a daily concern of crucial importance in the lives of these professionals. For this reason, many of them adopted measures to protect themselves and their families, leaving their homes, for instance, and staying in hotels^1^^,^ 7. In this critical period, the challenge was to provide care to individuals with COVID-19 and remain healthy to care for their families without contaminating them, so many opted for preventive isolation^1^^,^ 7, something unusual among the various challenges nurses experience.

Additionally, many nurses witnessed patients dying without the presence of their families and often mediated the contact, or farewell of severe patients with their families using a mobile phone or tablet, as it happened in different countries^21^^-^ 23, which impacted most participants in terms of the Fear dimension, but potentially more severely impacted those with a spouse or partner.

Psychological support became, even, more crucial as the workers had to deal with the finitude of patients, which was coupled with the isolation measures imposed, with the fact that the health conditions of patients would possibly worsen, and the fear that family members would be infected^24^. The central aspects of this pandemic context include providing support to spiritual needs^22^ including support provided to the health workers providing care to patients hospitalized because of the COVID-19.

In this study, specialist nurses stood out because they most frequently hold an Approach Acceptance attitude. Additionally, the nurses’ skills and knowledge are elements that promote different attitudes in care practice, resulting in positive strategies and behavior to deal with the disease^25^.

The probability of experiencing a large number of deaths is directly proportional to the number of people with COVID-19 to whom one provides care, consequently, a higher likelihood of dealing with death or the severe clinical condition of patients^7^. The surgical unit is characterized by the delivery of care intended to heal and treat patients so that dealing with death is not a daily or common experience in this unit; perhaps, for this reason, Avoidance and Fear of death are the most likely attitudes. However, when it became a unit where care is provided to patients with COVID-19, the experience of suffering and death became closer^7^, leading to the emergence of an Escape attitude.

Note that the front line workers have dealt with work overload given the intense demand for care, exacerbated by precautionary measures necessary to prevent the disease’s spread and avoid self-contagion. Furthermore, even in this challenging context, these workers provide emotional support to those hospitalized^23^. Additionally, the growing number of deaths leads to exhaustion and may also change attitudes and perceptions toward death.

One study^17^ reports that the process of mourning, death, and dying are unique experiences of each individual and cannot be standardized so that the signification of loss in times of pandemic is something complex and subject to change. Studies addressing this topic outside the unique context imposed by the COVID-19 have already warned that all elements in the professional context may influence the way nurses cope with death and how well this relationship can be explained^16^.

These results, especially those related to terminality, death, and mourning processes, are relevant for other countries recording even a more significant number of deaths, considering the possibility that the number of cases may increase again in the future and the potential occurrence of new pandemics^18^^-^ 19. These findings are also relevant for managers and institutions considering support measures to implement among workers dealing with death in health services and to guide personalized monitoring and support to nurses.

The fact that Fear and death Avoidance is more evidenced in a pandemic context reinforces the need to invest in the qualification of Nursing workers to cope with the death of those they provide care to, simultaneously minimizing the adverse effects these experiences may cause. Additionally, it is essential to ensure workers have specialized assistance to minimize the psychological distress to which they are exposed, aggravated by the pandemic context and reflect upon other aspects that require investments in particular situations as this and, among which, those related to the organization of work and working conditions.

Knowledge concerning nurses’ attitudes toward death within a hospital setting after the critical period of the COVID-19 pandemic enables understanding how the problem impacts the workers’ strategies to cope with such phenomena. It also enables to sharply signal their attitudes in the context of patients’ finitude, an aspect that is seldom addressed in undergraduate programs or training programs offered in health services. These findings, also, provide personalized data regarding the nurses’ profiles, which interfere in their attitudes toward death, showing managers and institutions the need to identify singularities when dimensioning the personnel to provide care in units where death is more prevalent among patients and the need to provide different types of inter-professional support, which can qualify care and the interaction with patients facing the process of death and dying, individualizing Nursing care.

The temporality of the data collected constitutes a limitation, suggesting the need for future studies addressing other hospital facilities post-pandemic. Additionally, because this study is part of a larger investigation and aware of the possibility of other approaches such as statistical methods of multiple analysis, the research team opted in the inferential stage to perform analyzes not conducted by any other study integrating this more extensive investigation. The statistical analysis performed did not allow assessing the influence of potential confounding variables.

## Conclusion

This study derives from a larger investigation that has analyzed the experience of nurses toward death and this stage is dedicated to analyzing the hospital context after the critical period of the COVID-19 pandemic in Portugal. Neutral/Neutrality Acceptance and Fear attitudes predominated and were influenced by marital status, profession, and work unit. The nurses directly providing care to patients hospitalized due to the COVID-19 during the pandemic’s critical period obtained the lowest mean in the Escape Acceptance dimension.

This study’s results can support institutions regarding coping strategies when facing pandemic and catastrophic events, in which strategies are needed to prepare workers to cope with death. These findings also provide information about the health of Nursing workers, considering that the pandemic impacts the physical health of workers and has repercussions on their attitudes and psychological aspects.
